# Comparative and Correlational Evaluation of the Phytochemical Constituents and Antioxidant Activity of *Musa sinensis* L. and *Musa paradisiaca* L. Fruit Compartments (Musaceae)

**DOI:** 10.1155/2020/4503824

**Published:** 2020-08-06

**Authors:** Barnabas Oluwatomide Oyeyinka, Anthony Jide Afolayan

**Affiliations:** Medicinal Plants and Economic Development (MPED) Research Centre, Department of Botany, University of Fort Hare, Alice 5700, South Africa

## Abstract

Secondary metabolites and their biological activity have pharmacological relevance in the prevention and therapeutic management of disease, including the facilitation of normal physiological processes through biochemical mechanisms. In this study, phytochemical constituents and antioxidant activity were evaluated quantitatively on the acetone, ethanol, and aqueous extracts of the flesh, and peel, as well as the boiled peel extract compartments of *Musa sinensis* L. and *Musa paradisiaca* L. fruits. Total phenol, proanthocyanidin, and flavonoid contents were estimated and measured spectrophotometrically. The free radical scavenging antioxidant capacity of the extracts was tested on DPPH (2,2-diphenyl-1-picrylhydrazyl ethanol), ABTS (2,2′-azino-bis(3-ethylbenzothiazoline-6-sulphonic acid)), and FRAP (ferric reducing antioxidant power) assay models. Correlation between phytoconstituents and antioxidant activity was analysed using Pearson's coefficient. The results showed varying amounts of phytochemicals in the solvent extracts of the flesh and peel, including the boiled peel extract of *M. sinensis* and *M. paradisiaca*. All acetone extracts of *M. sinensis* flesh, *M. paradisiaca* flesh, and *M. paradisiaca* peel had the highest phytochemical contents, with the exception of the ethanol extract of *M. sinensis* peel which had the highest phenol content; just as on the overall scale, the peel compartments had generally higher phytochemical profiles than the soft flesh in both fruits. The boiled peel extracts of *M. sinensis* and *M. paradisiaca* had the highest ABTS (0.03 mg/mL) and DPPH (0.03 mg/mL) activity. Ferric reducing power (FRAP) was the highest in the ethanol extracts of *M. sinensis* flesh and peel, and *M. paradisiaca* flesh, while it was the highest in the acetone extract of *M. paradisiaca* at the peak concentration used (0.1 mg/mL). There was a significant negative correlation between the total phenol and flavonoid contents of *M. sinensis* flesh with its DPPH radical scavenging activity and proanthocyanidin content of *M. paradisiaca* flesh with its DPPH radical scavenging activity. The correlation outcomes indicate that none of the phytochemical constituents solely affected antioxidant activity; instead, a combination of the polyphenolic constituents contributed to antioxidant activity. This study shows the therapeutic potentials of the flesh and, importantly, the peel of *M. sinensis* and *M. paradisiaca* fruits on the basis of the polyphenolic constitution against free radicals and oxidative stress.

## 1. Introduction

The medicinal relevance of plants chiefly lies in their chemistry, and plant secondary metabolites, in particular, are the key natural products that essentially drive the pharmacological activities of plants. Their utility spans industrial production frontiers such as pharmaceuticals, nutraceuticals, and textiles. Medicinal plants are so regarded because they possess therapeutic properties and have active components useful for drug synthesis and relevant in phytomedicine. Alkaloids, quinones, coumarins, and flavonoid compounds have been implicated in pharmaceutical processes [1] and the prominence of herbal medicine in many underdeveloped and developing countries. Scientific articles report that plant extract used in phytomedicine has paved the way for improved therapeutic scientific insights towards the development of the empirical medicinal system [2]. Oxidative stress, which in principle alters the physiological dynamics and triggers equilibrium disruption, is linked to series of inflammatory diseases such as arthritis, gastric ulcers, vasculitis, and lupus erythematous, including protein and DNA damage. In this regard, antioxidants provide defence mechanism through the prevention of oxidative damage, which is a notorious tendency of free radicals [2]. These defence mechanisms are expressed through enzymatic and nonenzymatic processes, some of which include the peroxidase, polyphenol oxidase, and the glutathione in its reduced form [3]. Antioxidants are peculiar in action because they have the capacity for repair, suppression of free radical formation, and scavenging. In biological systems, antioxidants express their mechanism through chelation of metal ion, regulation of gene expression, and the coantioxidants. Fruits are sources of many nonenzyme antioxidants like vitamins, gallic acid, rutin, and quercetin [4, 5].

Phytochemical compounds are secondary metabolites naturally sourced from fruits, vegetables, and other plant products, amongst them are the phenolics, which are central to the signal and defence mechanism in plants. Polyphenols help in reducing the risk of neurodegenerative disease, leukaemia, vasorelaxation, and antiallergenic activity [6] as well as possessing the capacity to interact with antioxidant enzymes [7, 8] such as glutathione S-transferases and the NADPH: quinine oxidoreductase (NQO1) and preventing the initiation phase of carcinogenesis through the modulation of cytoprotective enzyme activation [9]. The significance of dietary phytochemicals has been put forward in terms of being responsible for distinct plant characters like aroma and colour pigmentation [10]. It has also been reported that several phytochemicals are integral in affecting cell proliferation and regulation of cell cycle [10]. They are active in a series of signalling pathways that are usually disrupted during tumour initiation and proliferation [11–13].

Banana and plantain, members of the Musaceae family (*Musa* spp.), are important food crops that supply energy-based carbohydrates, including a wide spectrum of other nutritive components in human nutrition [14–16]. They are tropical herbaceous plants that grow up to heights reaching 9 metres, and are produced largely in the Asian, African, and South American regions; sweet fruits in the case of banana and for plantain are popularly cooked for food [17]. These plants have been implicated in agricultural and industrial uses, which make them valuable to the bioeconomy [18, 19]. Banana and plantain fruits possess very thick coverings known as the peels; however, they have low dietary incorporation status as they constitute waste because they are usually disposed during the consumption of the fruit pulp. It is with the foregoing that this study has been designed to evaluate polyphenolic constituents and biological activity of extracts across the compartments (flesh, peel, and boiled peel extract) of *M. sinensis* and *M. paradisiaca* fruits, with an aim to establish the phytomedicinal viability of the often neglected peel and boiled peel extract component. In so doing, this study seeks to project the peels of *M. sinensis* and *M. paradisiaca* as potential functional foods with medicinal, therapeutic, and nutritional value. With the foregoing, this study has been designed for the evaluation of the pharmacological potential in the extracts of *M. sinensis* and *M. paradisiaca* fruits, which will further validate the dietary medicinal value of the soft flesh and particularly the less utilised peels of the fruits.

## 2. Materials and Methods

### 2.1. Materials and Reagents

*M. sinensis* and *M. paradisiaca* fruits were obtained for this study from supermarkets in Alice and East London, located in the Amathole District Municipality, Eastern Cape Province, South Africa (latitude 32°43′28.66″; longitude 26°34′5.88″), and were authenticated by Professor C. N. Cupido, a taxonomist in the Botany Department, University of Fort Hare (UFH); afterwards, voucher specimens were deposited in the herbarium (UFH-2019-11-001 and UFH-2019-12-002).

Analytical grade reagents were used for the phytochemical and antioxidant experimental assays, and they include the Folin-Ciocalteau's (FC) reagent, aluminium trichloride (AlCl_3_), anhydrous sodium carbonate (Na_2_CO_3_), sodium hydroxide (NaOH), sodium nitrite (NaNO_2_), sodium acetate, hydrochloric acid (HCl), 2,2-diphenyl 1-1-picrylhydrazyl (DPPH), 2,2′-azino-bis (3-ethylbenzthiazoline-6-sulfonic acid) (ABTS), potassium persulfate (K_2_ ,S_2_ ,O_8_ ), ferric chloride (FeCl_3_), vanillin, rutin, trichloroacetic acid (TCA), quercetin, catechin, gallic acid, and methanol.

Pulverization of soft flesh and peel samples was done with a food blender (HBF 500S series, Virginia, USA). The boiling of peel samples was done in a water bath (BUCHI B-480). Filtration of sample-solvent mixtures was done using a set-up of the Whatman filter paper, Buchner funnel, and vacuum pump. Aqueous extracts were chilled in a refrigerant chiller (PolyScience AD15R-40-A12E, USA). Freeze-drying of aqueous extracts was done with a freeze dryer (Ceramic Filter Core Drier CD 052), Oven-drying of soft flesh and peel samples was done in an oven (LABOTEC, Durban, South Africa). Homogenization of extracts was done on a shaker (Lasec Stuart Orbital Shaker SSL1); organic (acetone and ethanol) extracts were concentrated using a rotary evaporator (LABOROTA 4000 Heidolph). Absorbance spectra were measured with Hewlett Packard VR-3000 PC spectrophotometer, 765 nm.

### 2.2. Sample and Extract Preparation

The fruits were well rinsed with distilled water, and the sliced soft flesh and the peel samples were oven-dried (LABOTEC, South Africa) at 40°C for 72 hours, after which the dried samples were pulverised using a food blender (Hamilton Beach HBF 500S Series). Another group of *M. sinensis* and *M. paradisiaca* peels (1000 g/L) were boiled in distilled water in the water bath (BUCHI B-480) at 80°C for 20 minutes. The boiled peel extracts were freeze-dried for 48 hours (Ceramic Filter Core Drier CD 052) and stored at 4°C until analytical work commenced. 150 g of the pulverised sample was weighed into three conical flasks, each containing 1,000 mL of acetone, ethanol, and distilled water solvents. Afterwards, the mixtures were homogenised constantly at 124 rpm on a shaker (Lasec Stuart Orbital Shaker SSL1) for 48 hours. The solvent mixtures were then pressure-filtered through the Whatman filter paper (No 1, 25 mm) in a Buchner funnel set-up on the vacuum pump. The acetone and ethanol filtrates were then concentrated further to obtain crude extracts using a rotary evaporator (LABOROTA 4000 Heidolph) at 56°C and 78°C [20–22] boiling points, respectively, while the aqueous filtrates were chilled in a refrigerant chiller (PolyScience AD15R-40-A12E, USA) at −40°C and concentrated to dry form using the freeze dryer for 48 hours (Ceramic Filter Core Drier CD 052). Water (aqueous) and ethanol extracts were prime organic solvent options since the medium of cooking and preparation of herbal medicines are infusion or decoction using water or alcohol, with acetone chosen because of its extractive ability for hydrophilic and lipophilic plant components. The extraction yields of the three solvents for the fruit compartments of *M. sinensis* and *M. paradisiaca* were evaluated and recorded. The extracts were stored at 4°C until further analysis.

### 2.3. Phytochemical Profiling

#### 2.3.1. Total Phenolic Content

Total phenol was determined using Folin-Ciocalteau's reagent by the method described by [23] with slight modifications to the concentration. A 0.5 mL aliquot of the different extracts (1 mg sample/mL in methanol) and the gallic acid standard was mixed with 2.5 mL of 10% (v/v) Folin-Ciocalteau's reagent and incubated for five minutes. To the solution, 2 mL of 7.5% (w/v) anhydrous sodium carbonate (Na_2_CO_3_) was added. The mixtures were incubated at 40°C for 30 minutes (LABOTEC-South Africa). Spectrophotometric measurement of absorbance was taken at 765 nm (Hewlett Packard VR-3000 PC spectrophotometer). Total phenolic (TP) content (mg GAE/g) was derived from the standard curve equation: *y* = 8.3093*x* − 0.0023, *R*^2^ = 0.9992. This was expressed from the formula *y* = *CV*/*m*, where *C* is the total content of phenolic compounds in plant extract in mg GAE/g, *V* is the volume of plant extract used in the assay in mL, *m* is the mass of extract used in the assay in g, and *y* is the concentration obtained from the standard curve in mg/mL.

#### 2.3.2. Total Proanthocyanidin Content

This was determined based on the method described by [24] with slight modification. 0.5 mL of extracts and different concentrations (0.0625 mg/mL to 1 mg/mL) of catechin (standard) were dispensed into different test tubes. Then, 3 mL of vanillin-methanol (4% w/v) and 1.5 mL of hydrochloric acid were added. The mixture was then vortexed and left to stand for 15 minutes at room temperature. The absorbance was spectrophotometrically measured at 500 nm (UV-3000 PC spectrophotometer). Total proanthocyanidin (TPR) content was evaluated using the equation of the calibration curve: *y* = 1.1493*x* + 0.0334, *R*^2^ = 0.9839, and it was expressed as mg catechin equivalent (CE)/g using the formula, *C* = *cV/m*, where *C* is the total proanthocyanidin content in plant extract in mg CE/g, *c* is the concentration obtained from the standard curve (mg/mL), *V* is the volume of plant extract used (mL), *m* is the mass of extract used (g).

#### 2.3.3. Total Flavonoid Content

Total flavonoid content in the extracts was determined using the colorimetric aluminium chloride method described by [25]. 0.5 mL of plant extracts and varying concentrations of quercetin standard, ranging from 0.0625 to 1.0 mg/mL, were dispensed into different test tubes. Into tubes were added 2 mL of distilled water and 0.15 mL of 5% sodium nitrite (NaNO_2_), with the solution made to stand for six minutes, after which 0.15 mL of 10% aluminium chloride (AlCl_3_) was added and incubated for five minutes. The mixtures were vortexed and incubated at 25°C for 15 minutes (LABOTEC, Durban, South Africa), and 1 mL of 1M sodium hydroxide was added in each tube. The solution was made up to 5 mL with the addition of 1.2 mL distilled water. Absorbance was measured at 420 nm with a spectrometer. Flavonoid content was quantitatively derived using the equation of the calibration curve:

(1) $$\begin{array}{c} y=1.1829x+0.0399,R^{2}=0.9929\text{ with formula }C=\frac{cV}{m}, \end{array}$$

where *C* is the total content of flavonoid compound in plant extract (mg QE/g), *c* is the concentration obtained from standard curve (mg/mL), *V* is the volume of plant extract used (mL), and *m* is the mass of extract used (g).

### 2.4. Antioxidant Activity

The antioxidant capacity of the fruits (flesh, peel, and boiled peel extract) of *M. sinensis* and *M. paradisiaca* was evaluated using the DPPH, ABTS, and the FRAP assays.

#### 2.4.1. DPPH Radical Scavenging Activity

DPPH radical scavenging activity was determined as described by [26]. Briefly, 1 mL of 0.135 mM DPPH radical (prepared with methanol in an opaque bottle) was added to 1.25 mL of the extracts and standards (gallic acid and rutin), each at varying concentration levels (0.0025 mg/mL to 0.01 mg/mL). The solution was vortexed and kept in dark condition at 25°C for 30 minutes. Absorbance was spectrophotometrically measured at 517 nm with methanol as blank. Scavenging activity was evaluated as follows:

(2) $$\begin{array}{c} \%\text{ DPPH inhibition}=\frac{\text{Abs}\left(\text{ control}\right)-\text{Abs}\left(\text{ 6sample}\right)}{\text{Abs}\left(\text{ control}\right)}\times 100\%, \end{array}$$

where Abs (control) is the absorbance of DPPH radical + methanol, and Abs (sample) is the absorbance of DPPH radical + sample extract or standard.

#### 2.4.2. ABTS Radical Scavenging Activity

This was determined as described in [27]. The stock solution of ABTS was prepared by mixing 7 mM ABTS and 2.45 mM potassium persulfate (K_2_S_2_O_8_) (1 : 1) and was incubated in the dark at 25°C for about 18 hours in order to release its radicals (ABTS^+^). The solution was diluted further by the mixture of 1 mL ABTS^+^ with 50 ml methanol to obtain a working solution (absorbance 0.700 ± 0.0006 at 734 nm). Plant extracts and rutin standard solution of concentration (0.005 mg/mL–0.08 mg/mL) were reacted with 1 mL ABTS in a test tube and kept in dark condition for 7 minutes. The absorbance was determined at 734 nm against methanol (blank):

(3) $$\begin{array}{c} \%\text{ABTS inhibition}=\frac{\text{Abs}\left(\text{ control}\right)-\text{Abs}\left(\text{ sample}\right)}{\text{Abs}\left(\text{ control}\right)}\times 100\%, \end{array}$$

where Abs (control) is the absorbance of ABTS radical +methanol  and Abs (sample) is the absorbance of ABTS radical +sample  extract/standard.

#### 2.4.3. FRAP Assay

The ferric reducing power of *M. sinensis* and *M. paradisiaca* extracts was determined using the method described by [28]. The FRAP reagent was prepared upon the mixture of 2.5 mL 0.2 M phosphate buffer reagent (62.5% monobasic + 37.5% dibasic; pH 6.6) and 2.5 mL of 1% potassium ferricyanide (K_3_Fe(CN)_6_). 1 mL of the standard rutin and plant extracts at varying concentration levels (0.025 mg/mL–0.1 mg/mL) were added to the FRAP solution. This mixture was incubated in a water bath (BUCHI B-480) at 50°C for 20 minutes. Afterwards, 2.5 mL of 10% trichloroacetic acid (TCA) was added and the mixture was centrifuged at 300 rpm for 10 minutes; then, 0.5 mL 0.1% iron (III) chloride (FeCl_3_), 2.5 mL of distilled water, and the 8.5 mL supernatant from the centrifuged solution (1 : 5:5) were mixed and incubated for 10 minutes, at room temperature. The ferric reducing ability of plant extracts (mg/g Fe (II) equivalent) was measured at 700 nm against the methanol blank. Ferric reducing power (iron (II)) was quantitatively derived using the following equation:

(4) $$\begin{array}{c} y=21.277x+0.6385,R^{2}=0.9988, \end{array}$$

and *C* = *c* × *V*/*m*, where *C* is the Fe (II) content of the FRAP solution in the plant extract (RE/g), *c* is the concentration of standard obtained from the standard curve (mg/mL), *V* is the volume of plant extract used (mL), and *m* is the mass of extract used (g).

### 2.5. Statistical Analysis

Data obtained from this study were expressed as mean ± standard deviation, based on triplicate experimentation. The statistical analysis carried out was done using the one-way analysis of variance (ANOVA) and mean separation by Fischer's LSD on the MINITAB statistical package.

## 3. Results and Discussion

Table 1 shows the extraction yield of the solvents from the fruit compartments of *M. sinensis* and *M. paradisiaca*. The aqueous and ethanol extracts had the highest and lowest yields, respectively, across the flesh and peel compartments of *M. sinensis* and *M. paradisiaca*. In this regard, a consistent yield trend in the order aqueous > ethanol > acetone was observed. On the other hand, boiled peel extracts (Be and Pe) had the lowest yields in their respective aqueous extract groups of *M. sinensis* and *M. paradisiaca*.

### 3.1. Phytochemicals

Table 2 shows total phenol, proanthocyanidin, and flavonoid contents of the acetone, aqueous, and ethanol solvent extractions of *M. sinensis* and *M. paradisiaca* fruits. In *M. sinensis*, the ethanol extract of the peel contained the highest phenol (157.19 ± 4.76 mg GAE/g), while the acetone extract of the peel also contained the highest proanthocyanidin (619.39 ± 21.88 mg CE/g) and flavonoid (734.52 ± 32.55 mg QE/g) contents. Similarly, in *M. paradisiaca*, the acetone extract of the peel contained higher phenol (136.87 ± 5.69 mg GAE/g) and proanthocyanidin (530.06 ± 53.51 mg CE/g) contents, while the acetone extract of the flesh contained the higher flavonoid (777.35 ± 150.95 mg QE/g) content. The boiled peel extract sample of *M. sinensis* contained the highest phenol (97.05 ± 3.60 mg GAE/g) and flavonoid (418.35 ± 427.26 mg QE/g) contents amongst the aqueous extractions, just as the boiled peel extract sample in *M. paradisiaca* had the highest phenol (114.23 ± 4.58 mg GAE/g) and flavonoid (83.58 ± 34.16 mg QE/g) contents. On the other hand, *M. paradisiaca* acetone extracts had the highest phenol (136.87 ± 5.59 mg GAE/g) and proanthocyanidin (530.06 ± 53.51 mg CE/g) in the peel with the highest flavonoid (777.35 ± 150.95 mg QE/g) in the flesh. Summarily, polyphenolic constituent was consistently predominant in the organic (acetone and ethanol) solvent extractions of *M. sinensis* and *M. paradisiaca* flesh and peel as evidenced by the extremely low or absent proanthocyanidin levels in all the aqueous extracts of *M. sinensis* and *M. paradisiaca* and for flavonoid in the aqueous extracts of *M. sinensis* flesh and peel and *M. paradisiaca* flesh as well.

### 3.2. Antioxidant Activity of the Acetone, Ethanol, and Aqueous Extracts of *M. sinensis* and *M. paradisiaca* Fruit Compartments

#### 3.2.1. DPPH

The percentage DPPH radical inhibitory activity of the *M. sinensis* extracts including standards (Table 3) was gallic acid > rutin > banana peel acetone extract > banana flesh ethanol extract > banana boiled peel extract > banana peel ethanol extract > banana flesh acetone extract > banana flesh aqueous extract > banana peel aqueous extract at the highest concentration (0.01 mg/mL).

For *M. paradisiaca*: gallic acid > rutin > plantain boiled peel extract > plantain peel aqueous extract > plantain peel acetone extract > plantain flesh ethanol extract > plantain peel ethanol extract > plantain flesh acetone extract > plantain flesh aqueous extract at the highest concentration (0.01 mg/mL).

Table 3 shows that the standard drugs (gallic acid (84.03 ± 0.002%) and rutin (64.12 ± 0.01%)) and all aqueous and ethanol extracts of the flesh and peel in *M. sinensis* and *M. paradisiaca* had peak antioxidant activity at the highest concentration gradient (0.01 mg/mL). A similar pattern existed in the acetone extracts of *M. sinensis* flesh (3.93 ± 0.02%) and peel (11.17 ± 0.002%) and *M. paradisiaca* peel (11.01 ± 0.002%).

#### 3.2.2. FRAP

Table 4 shows that ferric reducing power for banana group was in the order rutin > banana peel ethanol extract > banana peel aqueous extract > banana flesh ethanol extract > banana boiled peel extract > banana flesh acetone extract > banana flesh aqueous extract > banana peel acetone extract at the highest concentration (0.1 mg/mL).

The order for plantain was rutin > plantain peel acetone extract > plantain flesh ethanol extract > plantain peel ethanol extract > plantain flesh acetone extract > plantain boiled peel extract > plantain flesh aqueous extract > plantain peel aqueous extract at the highest concentration (0.1 mg/mL).

At the highest concentration gradient used (0.1 mg/mL), ethanol extracts of *M. sinensis* flesh (145.27 ± 1.94 mg/g) and peel (154.60 ± 1.47 mg/g) and *M. paradisiaca* flesh (153.81 ± 1.55 mg/g) had the highest ferric reducing power, while the acetone extract of *M. paradisiaca* peel (155.56 ± 1.81 mg/g) had the greatest ferric reducing power.

#### 3.2.3. ABTS

The ABTS radical scavenging activity of extracts shows that gallic acid had a generally higher scavenging activity than the rutin. The percentage ABTS inhibitory activity of the banana flesh and peel and peel extracts as well as the standards was of the following order: rutin > banana boiled peel extract > banana peel aqueous extract > banana peel ethanol > banana peel acetone extract > banana flesh acetone extract > banana flesh ethanol extract > banana flesh aqueous extract at the highest concentration (0.08 mg/L).

On the other hand, in plantain, the ABTS inhibitory activity order was rutin > plantain boiled peel extract > plantain peel aqueous extract > plantain peel ethanol extract > plantain peel acetone extract > plantain flesh ethanol extract > plantain flesh acetone extract > plantain flesh aqueous extract at the highest concentration level (0.08 mg/mL).

Table 5 shows that the rutin standard (99.10 ± 0.0004%) and acetone and ethanol extracts of *M. sinensis* and *M. paradisiaca* flesh and peel had the peak ABTS radical scavenging activity at the highest concentration gradient (0.08 mg/mL). However, amongst the aqueous extracts, all except *M. sinensis* and *M. paradisiaca* flesh had peak activity at the highest concentration gradient. *M. sinensis* flesh had its peak activity at 0.04 mg/mL, while *M. paradisiaca* had its own peak activity at 0.01 mg/mL concentration gradient.

Table 6 shows that peak activity in the plant extracts was observed in the acetone extract of *M. paradisiaca* flesh (<0.0025 mg/mL). However, ethanol and aqueous extracts of the flesh and peel, including the peel extract of *M. sinensis* and *M. paradisiaca*, had IC_50_ values above the highest concentration (0.01 mg/mL). From the IC_50_, ABTS had its peak activity in the peel extract of *M. sinensis* (0.03 mg/mL), followed by the aqueous extract of *M. sinensis* peel (0.04 mg/mL). On the other hand, *M. paradisiaca* had a joint peak activity in aqueous extracts of the peel and peel extract (0.04 mg/mL) (Table 6). The negative values observed in acetone and aqueous extracts of *M. paradisiaca* flesh (DPPH and ABTS) could suggest the preoxidant activity at particular concentration levels and potent scavenging capacity as well.

## 4. Correlation between Phytochemical Constituents and Antioxidant Activity of *M. sinensis* and *M. paradisiaca* Flesh and Peel Compartments

Pearson's coefficient was used to analyse the relationship existing between the pharmacological variables (total phenol, proanthocyanidin, and flavonoid) and antioxidant capacity (DPPH, ABTS, and FRAP) of the flesh and peel compartments of *M. sinensis* and *M. paradisiaca* fruits.

Table 7 shows a negative correlation between the respective phenolic, proanthocyanidin, flavonoid constituents, and the IC_50_ DPPH antioxidant capacity in *M. sinensis* flesh (*r* = −0.999; −0.938 and −0.998), *M. sinensis* peel (*r* = −0.123; −0.708 and −0.664), and *M. paradisiaca* flesh (*r* = −0.947; −0.998 and −0.979), while the IC_50_ DPPH antioxidant capacity of *M. paradisiaca* peel positively correlated with the proanthocyanidin (*r* = 0.43) and flavonoid (*r* = 0.323) constituents.

There was a negative correlation observed between the phenolic, proanthocyanidin, and flavonoid constituents and the IC_50_ ABTS antioxidant capacity of *M. sinensis* flesh (*r* = −0.962; −0.994 and −0.955), while positive correlation exists between the respective phytochemical constituents and the IC_50_ ABTS antioxidant capacity in *M. sinensis* peel (*r* = 0.919; 0.483 and 0.534), *M. paradisiaca* flesh (*r* = 0.840; 0.664 and 0.768), and *M. paradisiaca* peel (*r* = 0.203; 0.850 and 0.753) (Table 7).

There was negative correlation between the phenolic, proanthocyanidin, and flavonoid constituents and the IC_50_ FRAP antioxidant capacity in the flesh of *M. sinensis* (*r* = −0.092; −0.462 and −0.067), while the peel correlated positively (*r* = 0.799; 0.260 and 0.318). Conversely, in *M. paradisiaca*, the IC_50_ FRAP antioxidant capacity of the flesh correlated positively with the phenolic, proanthocyanidin, and flavonoid constituents (*r* = 0.690; 0.471 and 0.597), while the peel showed negative correlation (*r* = −0.866; −0.946 and −0.986) (Table 7).

Medicinal plants are widely used for their therapeutic potential which they derive from their array of bioactive principles. They are key in frontiers of natural products, green chemistry, and drug development [29].

Phenolics are secondary metabolites and are broadly dispersed in plant tissues. They are responsible for pigmentation, taste, and flavour in fruits [30]. A large number of phenols such as eugenol are responsible for taste [31]. Phenolic compounds have antioxidant activity which has stimulated nutritional interest [32]. The phenol content was high in all acetone and ethanol extracts of *M. sinensis* and *M. paradisiaca* which is similar to the phenolic levels detected in a number of medicinal plants such as *Terminalia arjuna*, *T. bellerica, T. chebula*, and *Phyllanthus emblica* [33]. Likewise, the observation is in line with the high phenolic content observed in *Morus nigra* cv. Cherokee, *Solanum melongena* cv. Blacknite, *Vaccinium corymbosum* cv. O'Neal, and *Capsicum annum* as reported by [34] just as [35] also reported similar phenolic content outcomes in the mesocarp and pericarp of *Mauritia flexuosa* and *Theobroma bicolor*.

Proanthocyanidins, also termed condensed tannins, are polymers of flavonoid molecules [36] present in fruits and flowers of several plants which function as a defence against biotic and abiotic stress and possess antitumor, antioxidant, and immunostimulatory capacity [37]. Furthermore, proanthocyanidins are hypoglycaemic agents reported to have free radical scavenging capacity [38]. They also have inhibitory activity against cancerous cells in the liver [39] and ovarian section [40]. Proanthocyanidin content is high in the ethanol extracts of *M. sinensis* and *M. paradisiaca* peels and is comparable to that of similar solvent extractants of *Brachylaena ilicifolia* [41]. In addition, the high proanthocyanidin contents in the acetone extracts of *M. sinensis* and *M. paradisiaca* fruits are similar to those detected in *Cucumis africanus* fruit by [42]. However, the low levels in the aqueous extracts are similarly corroborated by qualitative profiles of aqueous extracts of *M. sapientum* and *M. paradisiaca* and ripened Saba banana as well as in bracts of the Karpuravalli, Nendrum, and Poovan cultivars of banana [43–45].

Flavonoids are major secondary metabolites with extensive distribution in plants and pharmacological potentials [46]. They are non-nitrogenous compounds responsible for pigmentation in fruits, leaves, and petals [47], which are majorly sourced from fruits, vegetables, and cereals [48]. Flavonoids, known for their interaction with neurological signalling pathways [49], have therapeutic effects in neurodegenerative disease conditions. Dietary flavonoids have biochemical anticancer mechanisms by making the aryl hydrocarbon receptor (AhR) the target site [50] and similarly in Alzheimer's disease (AD) and atherosclerosis [51, 52] and chemoprevention and disease therapy [53]. They are also key in reducing the risk of chronic conditions and the improvement of blood pressure [54]. Notably, [55] has elucidated the use of dietary flavonoids in alternative medicine in relation to the control and management of epileptic conditions. Flavonoid content was high in the acetone and ethanol extracts of *M. sinensis* and *M. paradisiaca* flesh and peel, including the boiled peel extract in both fruits, but was the highest in the acetone extract of each of the flesh and peel of both fruits, a similar observation in ethyl acetate extracts of the muli cultivar of banana [56]. There was undetected to fairly low flavonoid content in the aqueous extracts of *M. sinensis* and *M. paradisiaca* in this study. However, the flavonoid content in *M. paradisiaca* boiled peel extract in this study is comparable to the observation in the aqueous extract of *Rubus niveus* fruit [57] and is also comparable to similar pharmacological profiles such as antiproliferative, antioxidant, and enzyme inhibitory activities of *Rubus caesius* L. [58, 59]. Generally, the higher phytochemical contents detected in the acetone and ethanol extracts over the aqueous extracts of *M. sinensis* and *M. paradisiaca* fruits suggest the superior extractive potency of organic solvents over the somewhat weaker potency in aqueous solvents [60]. This observation, particularly in acetone solvents, is not far-fetched due to the nature of the solvent's polarity, with extensive significance on the components and activity of bioactive compounds [61]. Furthermore, [62] has buttressed the point about organic solvents' high extraction efficiency.

Antioxidants are compounds that neutralise free radicals and can be supplied in diets. Dietary antioxidants have key pharmacologically important roles such as male reproductive health improvement [63], insulin resistance, and lowered risk of type II diabetes [64]. Essentially, antioxidants facilitate regular body functions such as normal cell growth, immune support, and prevention of degeneration at molecular levels, including the stemming of premature ageing [65]. Furthermore, research workers have mirrored areas such as the *in vitro* evaluation of the biological activities of unripe *M. paradisiaca* against the oxidative and cell-damaging phenomenon of lipid peroxidation in the pancreatic organ of rats [66]. Antioxidant potential was evaluated with the DPPH, ABTS, and FRAP assays, in order to account for the variances present in the mode of activity of antioxidants. There was a generally low DPPH activity in the acetone, ethanol, and aqueous extracts of the flesh and peel, as well as the boiled peel extracts in *M. sinensis* and *M. paradisiaca*. This could be accounted for due to the reported direct relation between DPPH radical scavenging activity and proanthocyanidin content [67]. Inhibition of ABTS^+^ (%) by the extracts was generally higher than in DPPH, alluding to a lower IC_50_ value than DPPH, which is explainable by the variations in the mechanism in the two radical antioxidant reaction assays. The organic extracts (acetone and ethanol) predominantly had the highest DPPH scavenging activity in the peel, flesh, and boiled peel extract of *M. sinensis*. This observation can be attributed to the implication of phenolic content in the antioxidative mechanism [68]. The synergistic relationship between phenolic compounds and antioxidant activity in plants has also been elucidated [69–71]. Reference [72] similarly observed good antioxidant scavenging activity in the peel of *Solanum tuberosum*, *Zingiber officinale*, *Ipomoea batatas*, and *Raphanus sativus*. ABTS inhibition was the highest in aqueous extracts of *M. sinensis* and *M. paradisiaca* peels and was even higher in the boiled peel extracts of *M. sinensis* and *M. paradisiaca*. The high phenolic content can be reflective of the observed antioxidant activity, especially because phenols and especially flavonoids are bioactivity precursors [73–75] and key contributory factors to the antioxidative potential of plants [76, 77].

The FRAP assay is a well-known, reliable approach that measures ferric reducing ability in a redox-linked colorimetric reaction that reduces Fe^3+^ to Fe^2+^ [78–81]. The reflectively high FRAP activity on the basis of IC_50_ valuation in ethanol extracts of *M. sinensis* flesh and peel and *M. paradisiaca* flesh, acetone extracts of *M. paradisiaca* flesh and peel, and aqueous extract of *M. sinensis* peel and *M. paradisiaca* boiled peel extract can be linked to high flavonoid content such as that seen in the ethanol extracts of *M. sinensis* flesh and peel, acetone extracts of *M. paradisiaca* flesh and peel, and *M. paradisiaca* boiled peel extract. This confers the flesh and peel of both fruits as natural antioxidant sources and further connotes the contributory role of secondary metabolites of *M. sinensis* and *M. paradisiaca* in disease prevention [82]. The positive correlation between phenolic content and the ABTS activity in *M. sinensis* peel, *M. paradisiaca* flesh and peel, FRAP activity of *M. sinensis* peel, and *M. paradisiaca* flesh is in conformity with reports of phenolic compounds being related to antioxidant activity [83–85]. However, the general positive and negative correlational patterns indicate an unpredictable correlation between the two pharmacological factors and suggest that none of the polyphenolic constituents contributed exclusively to antioxidant activity, but instead that a combination of the polyphenolic constituents contributed to antioxidant activity [71, 86].

## 5. Conclusion

In a bid to further enhance the utility of the phytomedicinal potential of *M. sinensis* and *M. paradisiaca* fruits, this study has shown their therapeutic potentials on the basis of their polyphenolic constitution and richness, chief of which are flavonoids and phenol in the compartments (flesh and peel) of the fruits. The bioactivity and therapeutic potential of the plant extracts are also largely inclined to the organic-based extraction solvents, with the acetone extracts being dominantly bioactive and thus suggested to be the most pharmacologically preferred extraction solvent. The biological activity reflects the viability of *M. sinensis* and *M. paradisiaca* fruits as useful natural antioxidants, against oxidative stress which improves and buttresses the nutritional acceptability and medicinal capacities, respectively, of the flesh and peel components of these fruits. Furthermore, *M. sinensis* and *M. paradisiaca* flesh and peel should be incorporated into the human diet due to comparable ferric reducing antioxidant capacity with standard drugs such as rutin. There also portend higher therapeutic benefits upon the combined consumption of the three fruit components.

## Figures and Tables

**Table 1:** Extraction yield of solvents.

Solvents	Fruit compartments	Extraction yield (%)
Aqueous	Bf	7.13
Aqueous	Bp	7.00
Aqueous	Be	6.67
Aqueous	Pf	7.86
Aqueous	Pp	7.67
Aqueous	Pe	7.33
Acetone	Bf	1.86
Acetone	Bp	1.00
Acetone	Pf	2.00
Acetone	Pp	1.33
Ethanol	Bf	2.67
Ethanol	Bp	2.00
Ethanol	Pf	4.00
Ethanol	Pp	3.13

B: banana (*M. sinensis*); P: plantain (*M. paradisiaca*); f: flesh; p: peel; e: boiled peel extract.

**Table 2:** Total polyphenolic contents of extracts.

	Samples	Phenol (mg GAE/g) *R*^2^ = 0.9992	Proanthocyanidin (mg CE/g) *R*^2^ = 0.9839	Flavonoid (mg QE/g) *R*^2^ = 0.9929
Acetone	Banana flesh	119.05 ± 5.80^cd^	337.48 ± 13.16^de^	602.64 ± 3.65^ab^
	Banana peel	128.20 ± 5.73^bc^	619.39 ± 21.88^a^	734.52 ± 32.55^a^
	Plantain flesh	114.80 ± 1.49^de^	436.09 ± 36.44^c^	777.35 ± 150.95^a^
	Plantain peel	136.87 ± 5.69^b^	530.06 ± 53.51^b^	750.87 ± 55.61^a^

Aqueous	Banana flesh	43.83 ± 1.13^h^	−48.84 ± 8.19^g^	−59.57 ± 3.01^e^
	Banana peel	47.68 ± 2.14^h^	−55.22 ± 1.88^g^	−42.01 ± 1.19^de^
	Banana boiled peel extract	97.05 ± 3.60^f^	−11.14 ± 2.46^g^	418.35 ± 427.26^bc^
	Plantain peel	83.32 ± 4.38^g^	−23.90 ± 8.90^g^	17.64 ± 3.01^de^
	Plantain flesh	17.41 ± 0.17^i^	−79.00 ± 8.52^g^	−55.62 ± 2.07^e^
	Plantain boiled peel extract	114.23 ± 4.58^de^	−33.18 ± 1.88^g^	83.58 ± 34.16^de^

Ethanol	Banana flesh	107.41 ± 4.67^ef^	274.83 ± 16.38^e^	501.20 ± 50.95^abc^
	Banana peel	157.19 ± 4.76^a^	446.53 ± 87.39^c^	582.35 ± 0.69^ab^
	Plantain flesh	75.14 ± 0.55^g^	68.33 ± 7.42^f^	249.27 ± 13.33^cd^
	Plantain peel	133.42 ± 8.18^b^	405.35 ± 18.51^cd^	641.53 ± 14.76^ab^

Banana (*M. sinensis*); plantain (*M. paradisiaca*). Significantly different means (*p* ≤ 0.05) not sharing the same letter.

**Table 3:** DPPH radical scavenging activity (%) of extracts and standards.

Concentration (mg/mL)				
Solvent	Sample	0.01	0.005	0.0025
Acetone	Bf	3.93 ± 0.02^cd^	1.89 ± 0.01^cd^	1.49 ± 0.01^cd^
	Bp	11.17 ± 0.002^cd^	8.73 ± 0.00^cd^	8.50 ± 0.00^cd^
	Pf	3.85 ± 0.004^cd^	3.70 ± 0.01^cd^	4.33 ± 0.001^cd^
	Pp	11.01 ± 0.002^cd^	2.28 ± 0.001^cd^	5.90 ± 0.003^cd^

Aqueous	Bf	0.55 ± 0.003^d^	−11.96 ± 0.07^d^	−0.47 ± 0.004^d^
	Bp	0.08 ± 0.01^cd^	−1.43 ± 0.01^cd^	−3.85 ± 0.005^cd^
	Be	8.42 ± 0.01^cd^	3.07 ± 0.005^cd^	−0.16 ± 0.01^cd^
	Pf	0.24 ± 0.005^cd^	−0.16 ± 0.001^cd^	−1.18 ± 0.005^cd^
	Pp	11.09 ± 0.03^cd^	1.73 ± 0.005^cd^	2.83 ± 0.004^cd^
	Pe	17.54 ± 0.003^c^	9.28 ± 0.004^c^	8.26 ± 0.01^c^

Ethanol	Bf	9.91 ± 0.001^cd^	7.47 ± 0.002^cd^	6.84 ± 0.002^cd^
	Bp	5.27 ± 0.002^cd^	−0.47 ± 0.004^cd^	−3.07 ± 0.01^cd^
	Pf	10.46 ± 0.001^cd^	7.71 ± 0.004^cd^	5.66 ± 0.003^cd^
	Pp	6.61 ± 0.02^cd^	3.54 ± 0.01^cd^	−0.24 ± 0.005^cd^
	Gallic acid	84.03 ± 0.002^a^	70.81 ± 0.02^a^	51.38 ± 0.001^a^
	Rutin	64.12 ± 0.01^b^	35.09 ± 0.005^b^	15.42 ± 0003^b^

B: banana; P: plantain; f: flesh; p: peel; e: boiled peel extract. Banana (*M. sinensis*); plantain (*M. paradisiaca*).

**Table 4:** Ferric reducing power (mg RE/g) of the extracts and standard.

Concentration (mg/mL)				
Sample		0.1	0.05	0.025
Acetone	Bf	142.76 ± 2.02^ab^	147.09 ± 1.44^ab^	144.56 ± 1.32^ab^
	Bp	138.42 ± 1.46^ab^	148.71 ± 2.76^ab^	137.13 ± 0.64^ab^
	Pf	145.38 ± 3.25^ab^	140.09 ± 0.89^ab^	137.27 ± 3.36^ab^
	Pp	155.56 ± 1.81^a^	153.53 ± 1.42^a^	150.45 ± 1.38^a^

Ethanol	Bf	145.27 ± 1.94^ab^	146.09 ± 2.19^ab^	141.22 ± 2.02^ab^
	Bp	154.60 ± 1.47^a^	154.15 ± 1.10^a^	139.58 ± 5.64^a^
	Pf	153.81 ± 1.55^a^	150.82 ± 0.87^a^	143.82 ± 3.37^a^
	Pp	146.48 ± 1.40^ab^	140.84 ± 2.13^ab^	148.75 ± 3.22^ab^

Aqueous	Bf	141.55 ± 3.05^ab^	138.35 ± 0.78^ab^	144.53 ± 0.92^ab^
	Bp	147.10 ± 1.87^ab^	140.32 ± 2.48^ab^	135.48 ± 3.47^ab^
	Be	144.40 ± 2.40^ab^	137.92 ± 2.11^ab^	146.07 ± 2.08^ab^
	Pf	143.35 ± 1.91^ab^	139.42 ± 4.19^ab^	135.79 ± 1.48^ab^
	Pp	137.71 ± 1.83^ab^	137.60 ± 2.60^ab^	139.09 ± 2.61^ab^
	Pe	144.04 ± 0.58^ab^	138.33 ± 2.05^ab^	143.76 ± 5.02^ab^
	Rutin	195.95 ± 2.18^b^	101.44 ± 0.99^b^	47.25 ± 0.45^b^

B: banana; P: plantain; f: flesh; p: peel; e: boiled peel extract. Significantly different means (*p* ≤ 0.05) not sharing the same letter. Banana (*M. sinensis*); plantain (*M. paradisiaca*).

**Table 5:** ABTS radical inhibition activity (%) by extracts and standard.

Solvent	Sample	0.08 mg/mL	0.04 mg/mL	0.02 mg/mL	0.01 mg/mL	0.005 mg/mL
Acetone	Bf	37.56 ± 0.003^bcde^	30.09 ± 0.002^bcde^	25.17 ± 0.004^bcde^	24.38 ± 0.004^bcde^	21.93 ± 0.004^bcde^
	Bp	42.08 ± 0.001^bcde^	28.12 ± 0.01^bcde^	24.29 ± 0.004^bcde^	23.89 ± 0.01^bcde^	23.99 ± 0.004^bcde^
	Pf	29.20 ± 0.01^de^	24.29 ± 0.005 ^de^	15.34 ± 0.01^de^	15.53 ± 0.004^de^	11.80 ± 0.01^de^
	Pp	49.65 ± 0.004^abcd^	34.71 ± 0.001^abcd^	28.71 ± 0.003^abcd^	24.29 ± 0.004^abcd^	23.70 ± 0.005^abcd^

Aqueous	Bf	24.19 ± 0.003^cde^	24.58 ± 0.002^cde^	19.66 ± 0.001^cde^	18.19 ± 0.00^cde^	17.80 ± 0.002^cde^
	Bp	74.93 ± 0.004^abc^	47.79 ± 0.004^abc^	31.66 ± 0.006^abc^	23.20 ± 0.001^abc^	20.06 ± 0.004^abc^
	Be	87.90 ± 0.01^a^	57.62 ± 0.01^a^	37.95 ± 0.01^a^	26.06 ± 0.003^a^	31.37 ± 0.003^a^
	Pf	9.64 ± 0.01^e^	11.40 ± 0.005^e^	11.21 ± 0.004^e^	13.86 ± 0.001^e^	11.99 ± 0.01^e^
	Pp	54.28 ± 0.002^abcde^	51.72 ± 0.005^abcde^	24.19 ± 0.01^abcde^	20.25 ± 0.01^abcde^	0.69 ± 0.004^abcde^
	Pe	90.17 ± 0.04^ab^	47.10 ± 0.001^ab^	38.54 ± 0.004^ab^	19.47 ± 0.01^ab^	19.07 ± 0.004^ab^

Ethanol	Bf	33.82 ± 0.004^bcde^	24.19 ± 0.00^bcde^	24.88 ± 0.00^bcde^	21.73 ± 0.004^bcde^	23.01 ± 0.004^bcde^
	Bp	60.27 ± 0.003^abcd^	42.48 ± 0.002^abcd^	31.86 ± 0.004^abcd^	24.19 ± 0.01^abcd^	26.06 ± 0.004^abcd^
	Pf	30.28 ± 0.003^bcde^	27.04 ± 0.003^bcde^	22.42 ± 0.001^bcde^	21.04 ± 0.01^bcde^	19.37 ± 0.003^bcde^
	Pp	53.10 ± 0.003^bcde^	31.66 ± 0.003^bcde^	19.37 ± 0.002^bcde^	15.83 ± 0.003^bcde^	13.37 ± 0.001^bcde^
	Rutin	99.10 ± 0.0004^a^	80.33 ± 0.01^a^	31.37 ± 0.002^a^	18.68 ± 0.01^a^	11.70 ± 0.002^a^

**Table 6:** IC_50_ values (mg/mL) of extracts and standards.

	Extract/standard	DPPH		ABTS	
		IC_50_	*R* ^2^	IC_50_	*R* ^2^
Acetone	Banana flesh	0.08	0.8941	0.14	0.9904
	Banana peel	0.11	0.937	0.12	0.9377
	Plantain flesh	−0.92	0.3324	0.17	0.9243
	Plantain peel	0.06	0.5268	0.08	0.9971
Ethanol	Banana flesh	0.10	0.9811	0.20	0.8756
	Banana peel	0.05	0.9994	0.06	0.9873
	Plantain flesh	0.07	0.989	0.21	0.9457
	Plantain peel	0.06	0.9389	0.07	0.9976
Aqueous	Banana flesh	0.12	0.068	0.35	0.747
	Banana peel	0.11	0.8991	0.04	0.998
	Banana boiled peel extract	0.05	0.9977	0.03	0.9802
	Plantain flesh	0.30	0.8176	−0.91	0.6616
	Plantain peel	0.04	0.8176	0.06	0.7657
	Plantain boiled peel extract	0.03	0.9468	0.04	0.9795
	Gallic acid	0.001	0.9129	—	—
	Rutin	0.01	0.9937	0.03	0.9058

**Table 7:** Correlation between phytochemical constituents and antioxidant activity of *M. sinensis* and *M. paradisiaca* flesh and peel compartments.

Pearson's coefficient (r)			
Antioxidant parameters	Total phenol	Total proanthocyanidin	Total flavonoid
IC_50_ DPPH Bf	−0.999^*∗*^	−0.938^ns^	−0.998^*∗*^
IC_50_ DPPH Bp	−0.123^ns^	−0.708^ns^	−0.664^ns^
IC_50_ DPPH Pf	−0.947^ns^	−0.998^∗^	−0.979^ns^
IC_50_ DPPH Pp	−0.313^ns^	0.473^ns^	0.323^ns^
IC_50_ ABTS Bf	−0.962^ns^	−0.994^ns^	−0.955^ns^
IC_50_ ABTS Bp	0.919^ns^	0.483^ns^	0.534^ns^
IC_50_ ABTS Pf	0.840^ns^	0.664^ns^	0.768^ns^
IC_50_ ABTS Pp	0.203^ns^	0.850^ns^	0.753^ns^
IC_50_ FRAP Bf	−0.092^ns^	−0.462^ns^	−0.067^ns^
IC_50_ FRAP Bp	0.799^ns^	0.260^ns^	0.318^ns^
IC_50_ FRAP Pf	0.690^ns^	0.471^ns^	0.597^ns^
IC_50_ FRAP Pp	−0.866^ns^	−0.946^ns^	−0.986^ns^

ns: nonsignificant; significance (*p* < 0.05). Bf: banana flesh; Bp: banana peel; Pf: plantain flesh; Pp: plantain peel.

## Data Availability

The data used to support the findings of this study are available upon request to the authors.
